# Hypoxia Induced Heparan Sulfate Primes the Extracellular Matrix for Endothelial Cell Recruitment by Facilitating VEGF-Fibronectin Interactions

**DOI:** 10.3390/ijms20205065

**Published:** 2019-10-12

**Authors:** Jo Ann Buczek-Thomas, Celeste B. Rich, Matthew A. Nugent

**Affiliations:** 1 Department of Biomedical Engineering, Boston University, Boston, MA 02215, USA; jbuczek@bu.edu; 2 Department of Biochemistry, Boston University School of Medicine, Boston, MA 02215, USA; cbrich@bu.edu; 3 Department of Biological Sciences, University of Massachusetts Lowell, Lowell, MA 01854, USA

**Keywords:** age-related macular degeneration, angiogenesis, endothelial cell, fibronectin, hypoxia, retina, vascular endothelial growth factor

## Abstract

Vascular endothelial growth factor-A (VEGF) is critical for the development, growth, and survival of blood vessels. Retinal pigmented epithelial (RPE) cells are a major source of VEGF in the retina, with evidence that the extracellular matrix (ECM)-binding forms are particularly important. VEGF associates with fibronectin in the ECM to mediate distinct signals in endothelial cells that are required for full angiogenic activity. Hypoxia stimulates VEGF expression and angiogenesis; however, little is known about whether hypoxia also affects VEGF deposition within the ECM. Therefore, we investigated the role of hypoxia in modulating VEGF-ECM interactions using a primary retinal cell culture model. We found that retinal endothelial cell attachment to RPE cell layers was enhanced in cells maintained under hypoxic conditions. Furthermore, we found that agents that disrupt VEGF-fibronectin interactions inhibited endothelial cell attachment to RPE cells. We also found that hypoxia induced a general change in the chemical structure of the HS produced by the RPE cells, which correlated to changes in the deposition of VEGF in the ECM, and we further identified preferential binding of VEGFR2 over VEGFR1 to VEGF laden-fibronectin matrices. Collectively, these results indicate that hypoxia-induced HS may prime fibronectin for VEGF deposition and endothelial cell recruitment by promoting VEGF-VEGFR2 interactions as a potential means to control angiogenesis in the retina and other tissues.

## 1. Introduction

Vascular endothelial growth factor-A (VEGF) is a critical factor required for the development, growth, and survival of blood vessels [1,2]. Multiple forms of VEGF are produced through alternate mRNA splicing to generate at least four major isoforms in humans, as follows: VEGF_121_, VEGF_165_, VEGF_189_, and VEGF_206_ [2]. All of the major VEGF-A isoforms are secreted from cells as homodimers, and all except VEGF_121_ contain a consensus heparin-binding domain in the C-terminal region that is comprised of basic amino acids. VEGF has been implicated in a number of ocular diseases [3,4]. While many cell types produce VEGF, studies have indicated that retinal pigmented epithelial (RPE) cells are a major source of VEGF in the retina [5], with evidence that the high molecular weight extracellular matrix (ECM)-binding forms are particularly important [6]. The major tyrosine kinase receptors VEGFR1 (Flt-1) and VEGFR2 (KDR, Flk-1) are expressed on vascular endothelial cells (EC) as well as other cell types to varying degrees [3,7,8]. VEGF stimulates EC proliferation, migration, survival, and vascular permeability, with most of these activities being attributed to signaling through VEGFR2 and its tyrosine kinase activity. VEGFR1, and its soluble extracellular domain (sVEGFR1), on the other hand, is a kinase-impaired receptor whose major role appears to be to function as a decoy receptor [9,10].

Deposition of VEGF within the ECM was originally thought to be a means to sequester VEGF away from cell surface receptors and to stabilize gradients; however, several studies have revealed that VEGF associated with the ECM may mediate distinct signals in endothelial cells that are required for full angiogenic activity [11,12,13,14]. While ECM association of VEGF was generally believed to involve direct interactions between VEGF and heparan sulfate (HS), we and others have noted that the major binding site in the ECM for VEGF is fibronectin (Fn) [13,14,15,16]. This is particularly interesting considering the observations that Fn expression is increased in diabetes and that agents that reduce Fn have been shown to reduce retinal vascular permeability in diabetic rats [17,18,19]. However, HS remains a critical factor for this interaction as the major VEGF-binding site on Fn resides within the Hep 2 domain on Fn that is only available to bind VEGF after it has been modified by transient interactions with catalytically active HS [16,20,21]. Interestingly, we have also found that PDGF binds to this same site on Fn and have implicated this process in PDGF-driven cell movements during *xenopus* morphogenesis [22]. HS also plays critical roles on cell surfaces in mediating VEGF interactions with receptors, which appear to principally involve HS binding to VEGF-receptors and not direct binding of VEGF to HS as was previously thought [23,24,25]. Thus, HS appears to play central roles in modulating VEGF through mechanisms that are independent of its ability to directly bind VEGF. This is in contrast to better defined systems such as with the fibroblast growth factors where HS binds to the growth factor and its receptor to create a high affinity ternary complex [26,27]. As such, it is of particular interest to probe these mechanisms in more detail to understand what regulates the ECM’s capacity to bind VEGF and present it to endothelial cells.

A hallmark of insufficiently vascularized tissues is low oxygen tension, or hypoxia. As such, hypoxia has been implicated as a major driving force for angiogenesis, the growth of new blood vessels [28,29,30]. Hypoxia stimulates the expression of the transcription factor hypoxia-inducible factor 1α which leads to increased VEGF expression [28,30]. However, little is known about whether hypoxia also leads to changes that might affect VEGF deposition within an Fn-rich ECM. Therefore, we investigated the role of hypoxia in modulating VEGF-Fn interactions using a primary retinal cell culture model. We found that retinal endothelial cell attachment was enhanced to retinal pigmented epithelial (RPE) cell layers maintained under hypoxic conditions. Furthermore, our data indicate that this process was correlated with changes in VEGF, Fn, and HS proteoglycans. We found that hypoxia induced a general change in the chemical structure of the HS produced by the RPE cells, which correlated to changes in the amount and capacity of VEGF in the ECM, and we further identified preferential binding of VEGFR2 over VEGFR1 to VEGF rich-Fn matrices. Collectively, these results indicate that hypoxia-induced HS primes Fn within the extracellular matrix for VEGF deposition and endothelial cell recruitment by promoting VEGF-VEGFR2 interactions that may contribute to choroidal neovascularization, as well as angiogenesis, in other tissues.

## 2. Results

### 2.1. Endothelial Cell Attachment to Retinal Pigmented Epithelial Cells is Enhanced Under Hypoxic Conditions

**Figure 1:**
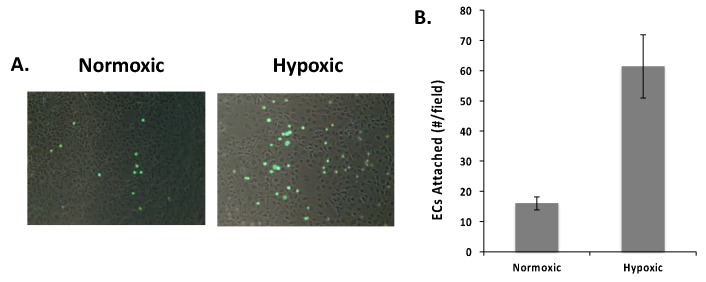
Hypoxia enhances endothelial cell attachment to retinal pigmented epithelial (RPE) cell layers. RPE cells were maintained under normoxic (20% pO_2_) and hypoxic (1% pO_2_) conditions for 48 h prior to cell attachment. Fluorescently labeled retinal endothelial cells (RECs) were allowed to attach for 1 h and fixed prior to visualization (**A**) by fluorescence microscopy and (**B**) counting (4 fields/well; 3 wells/condition; biological replicates (*N*) = 3; technical replicates (*n*) = 4). The data in panel (**B**) are expressed as the average number of cells per field ± SEM. Hypoxia caused a statistically significant increase in REC attachment (*p* < 0.05).

RPE cells have been identified as a major source of VEGF in the retina and previous studies have shown that the ECM binding form of VEGF plays a central role in the recruitment of choroidal endothelial cells to RPE cell layers [5]. Thus, it is possible that hypoxic conditions could enhance the endothelial cell recruitment activity of RPE cells. As an early step in endothelial cell recruitment, we evaluated the attachment of endothelial cells to RPE cells. For these studies, RPE cells were subject to normoxic (20% pO_2_) or hypoxic (1% pO_2_) conditions for 48 h. Retinal endothelial cells (REC) were fluorescently labeled with Vybrant DiO and allowed to attach to the RPE cell layers for 1 h prior to fixing and visualization by fluorescence microscopy, and the number of cells counted. As shown in Figure 1, we observed a dramatic increase in endothelial cell attachment to hypoxic RPE cell layers with respect to normoxic controls (62 vs. 16 cells per field respectively). To ensure that the increased number of RECs attached to the hypoxic RPE cultures was not simply the result of increased attachment to the underlying plastic dish, we conducted a visual analysis of each image to determine if each REC was on top of all or part of an RPE (cell) or between the RPE cells (plastic). Unless clear evidence of a portion of an RPE cell body, a nucleus, or nucleoli could be detected under a fluorescent REC, we “scored” the REC as being attached to “plastic”. From this analysis, we note that 68% and 75% of the attached endothelial cells were on top of the RPE cells in the normoxic and hypoxic conditions, respectively. Thus, the increased EC attachment observed with RPE cells that were subjected to hypoxia conditioning was reflective of an increase in the number of ECs attached to the cell layer and not a result of increased exposure and attachment of ECs to the underlying plastic dish. It is also important to note that these experiments were designed such that RPE cell density was at its maximum (i.e., longer growth or higher plating densities did not result in increased RPE cell density at the time of the EC attachment). Hence, cells that were “scored” as being attached to “plastic” were potentially attached to the extracellular matrix deposited by the neighboring RPE cells. As such, we included all attached cells in the analysis presented in Figure 1, and conclude that hypoxia caused the RPE cell layer to become more adhesive to ECs.

### 2.2. Hypoxia Enhances Endothelial Cell Attachment by Increasing ECM-Bound VEGF

**Figure 2:**
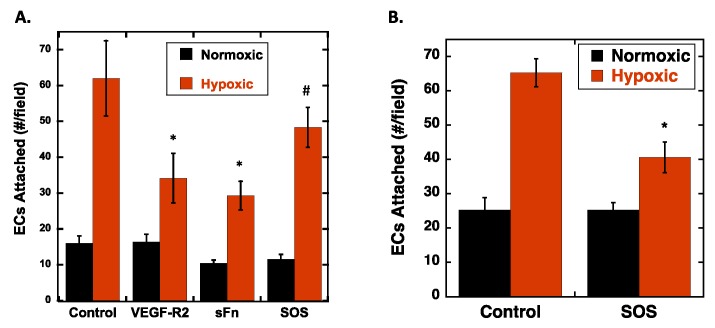
Soluble VEGF-ECM (vascular endothelial growth factor-A-extracellular matrix) Antagonists inhibit endothelial cell attachment to hypoxic RPEs. (**A**) RPE cells were subjected to normoxic (20% pO_2_) or hypoxic (1% pO_2_) conditioning for 48 h. Fluorescently labeled REC were allowed to attach in the presence of the indicated soluble effector: Fc-VEGFR2 chimera (100 ng/mL); soluble 40 kDa Hep2 domain of Fn (10 µg/mL); and sucrose octasulfate (SOS; 100 µg/mL). After the attachment period, the cells were washed, fixed, and counted (*n* = 4 fields/well; *N* = 3 wells/condition). The data is expressed as the average number of attached cells per field ± SEM. * ANOVA followed by Turkey’s tests revealed that hypoxic versus control, and Fc-VEGFR2 chimera and sFn on hypoxic cells versus hypoxic cells were statistically different (*p* < 0.05), ^#^ hypoxic control and SOS on hypoxic cells were not statistically different (*p* = 0.08). (**B**) SMC were subjected to normoxia or hypoxia for 48 h and REC were allowed to attach for 1 h in the presence and absence of SOS (500 µg/mL), and fluorescent cells counted as described above. Hypoxia significantly increased REC attachment to SMCs compared to normoxic cells, and (*) SOS significantly inhibited SMC attachment to hypoxic cells (*N* = 3; *n* = 4) (*p* < 0.05).

To determine if the increase in adhesion on cells subjected to hypoxia was related to increased VEGF in the ECM of the RPE cells, we conducted cell attachment experiments in the presence of various VEGF-ECM antagonists, as follows: Soluble Fc-VEGFR2 chimera that specifically binds to VEGF was used to block VEGF receptors on the REC from binding to VEGF in the ECM; a soluble fragment of Fn containing the Hep 2 domain, that binds to VEGF, was used to compete with VEGF localized to the ECM [21]; and sucrose octasulfate (SOS), a small charged disaccharide, was used to release VEGF from Fn in the ECM [31] (Table 1). Fc-VEGFR2 and sFn showed statistically significant inhibition of REC adhesion by 45%–55%. SOS inhibited attachment by 22%, which was not statistically significant (*p* = 0.08) (Figure 2A). While the partial reduction in REC attachment by SOS was not statistically significant, it is interesting to note that at the concentration of SOS used (100 µg/mL) we see a similar modest reduction in VEGF binding to Fn [31]. Unfortunately, higher concentrations of SOS, which would be predicted to release a greater fraction of ECM bound VEGF, caused RPE detachment and thus could not be used in these assays. However, a different cell type, rat vascular smooth muscle cells (SMC), was resistant to higher concentrations of SOS, thus experiments were conducted with these cells using 500 µg/mL SOS (Figure 2B). As with RPEs, hypoxic conditioning of SMCs resulted in increased attachment of RECs, and moreover, the presence of SOS at 500 µg/mL resulted in statistically significant (~40%) inhibition of REC attachment to the hypoxic SMCs. Taken together, these data suggest that increased cell adhesion to hypoxic cells was dependent, in part, on increased VEGF levels in the ECM.

**Figure 3:**
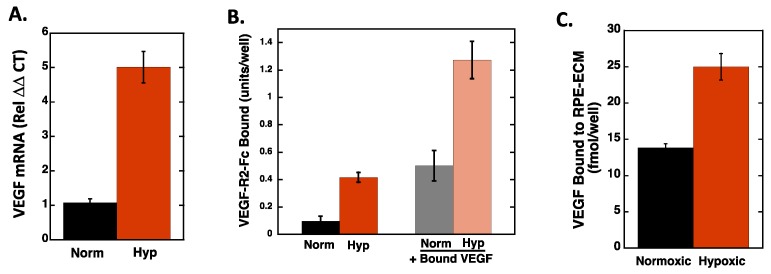
Hypoxia increases VEGF expression, VEGF deposition in the ECM, and VEGF binding in RPE cells. RPE cells were subjected to normoxia and hypoxia for 72 h. (**A**) Total RNA was extracted from RPE cells and mRNA quantified using qPCR analyses, as described in Materials and Methods. The data is normalized to the 18S RNA and to the normoxic control for each condition and expressed as the average ± SEM. (*N* = 4; *n* = 2). (**B**) After the normoxic/hypoxic conditioning, RPE cells were washed and incubated with and without 50 ng/mL VEGF for 1 h at 4 °C. Unbound VEGF was removed from the cells by washing and the cells were subsequently incubated with Fc-VEGFR2 Chimera. Bound Fc-VEGF-R2 was detected with an HRP-linked secondary antibody. The data are expressed as average background corrected absorbance values ± SD (*N* = 4). (**C**) ^125^I-VEGF was bound to normoxia/hypoxia were RPE cells and the amount bound to the ECM extracted and quantitated. Data represent the average ± SD of quadruplicate wells (*N* = 4). Hypoxia induced a statistically significant increase in VEGF mRNA (**A**), VEGFR2 binding (**B**), and VEGF increased VEGFR2 binding to both normoxic and hypoxic cells (**B**), and hypoxia increased VEGF binding to the ECM of RPE cells.

**Figure 4:**
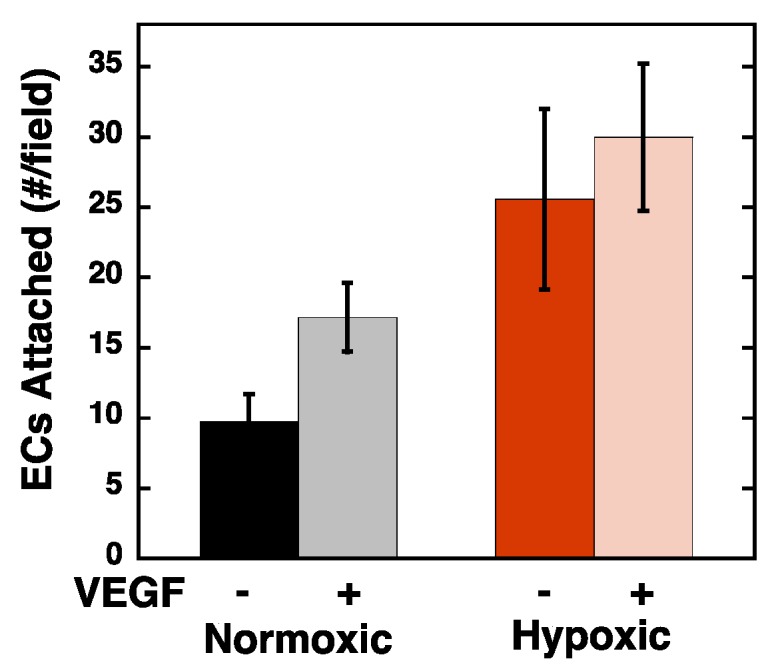
RPE cells were subjected to normoxia and hypoxia for 72 h. RPE cells were allowed to incubate with and without VEGF (50ng/mL), then washed, and fluorescently labeled RECs were allowed to attach for 1 h, washed, fixed, and quantified as described above (expressed as the average number of cells per field, *N* = 3; *n* =4; ±SEM). Hypoxia and VEGF significantly increased REC attachment as compared to normoxic cells (*p* < 0.05). VEGF did not significantly increase REC attachment to hypoxic RPEs.

To directly measure the impact of hypoxia on VEGF expression, quantitative real-time PCR was used to measure VEGF mRNA levels, and the Fc-VEGFR2 chimera was used in an ELISA format to measure VEGF presence on the RPE cell layer (Figure 3A,B). VEGF mRNA levels were increased ~5-fold, and Fc-VEGFR2 binding was increased ~4-fold in hypoxic compared to normoxic RPE cells. As an additional control, we also bound exogenous VEGF to the normoxic and hypoxic RPE cells and noted a corresponding increase in Fc-VEGFR2 binding, indicating that the ECM in the hypoxic cells has a higher capacity for VEGF binding than that in normoxic cells. Furthermore, we directly measured binding of ^125^I-VEGF to the ECM of RPE cells after hypoxic conditioning (Figure 3C) and noted an ~2-fold increase in the amount of VEGF bound. Similarly, when normoxic and hypoxic RPE cell/ECM layers were pretreated with VEGF, REC attachment to the normoxic RPEs increased in the presence of VEGF, indicating that bound VEGF can function to enhance RECs attachment (Figure 4). Interestingly, the addition of VEGF only slightly, and not significantly, increased REC attachment to the hypoxic RPEs indicating that the impact of VEGF on the RECs may approach saturation under these conditions.

### 2.3. VEGF Receptor 2 Binds to VEGF-Fibronectin

**Figure 5:**
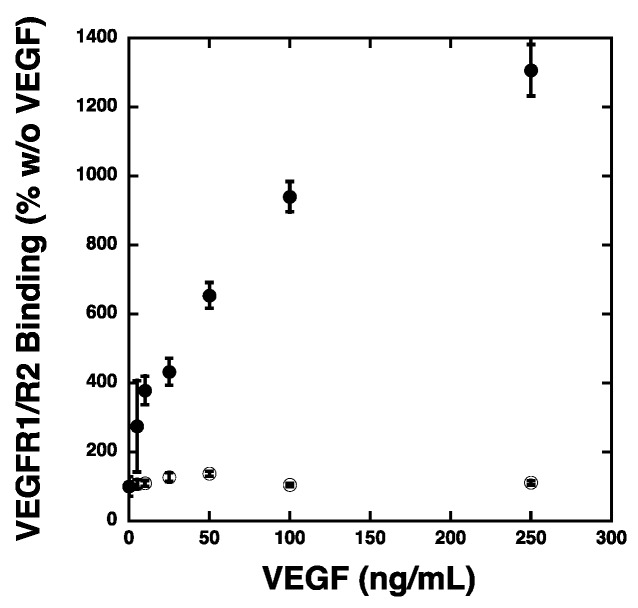
Fc-VEGFR2 binds to VEGF-Fn in a VEGF-Dependent manner. Fn (fibronectin) was adsorbed onto polystyrene plates overnight at 4 °C in the presence of heparin. The wells were washed to remove the heparin and incubated with the indicated VEGF concentrations. Unbound VEGF was removed prior to addition of 25 ng Fc-VEGFR1 (open circles) or Fc-VEGFR2 (closed circles) for 1 h at room temperature. The wells were washed and the Fc-chimera proteins were detected with HRP labeled goat anti human IgG. The absorbance values were determined in quadruplicate wells and the data is expressed as the average (*N* = 4) percentage relative to the absorbance observed with each chimera when no VEGF was present (zero VEGF was set to 100% for each chimera) ± SEM.

The ability of the Fc-VEGFR2 chimera to inhibit endothelial cell attachment and to bind to VEGF-bound onto RPE cultures suggest that VEGF receptors on the endothelial cells can bind to ECM-bound VEGF as a component of the mechanism of attachment. Thus, we evaluated the ability of VEGF receptors to bind to VEGF-laden Fn matrices. For these studies, Fn matrices were treated with heparin overnight at 4 °C to expose VEGF binding sites prior to allowing VEGF to bind to the matrix (Figure 5). The VEGF-matrices were incubated with Fc-VEGFR1 or Fc-VEGFR2 chimera proteins. The Fc-VEGF receptors that bound to the Fn matrices were detected using a secondary antibody that recognized the Fc region of the chimeric proteins. The data show little change in Fc-VEGFR1 association with the VEGF-fibronectin matrix over a wide range of VEGF concentrations. However, we observed that Fc-VEGFR2 association to VEGF-Fn was increased in a VEGF concentration dependent fashion. Importantly, the inability of the Fc-VEGFR1 to bind to VEGF-Fn was not the reflective of a physical deficiency of this chimeric protein as we have previously demonstrated that this reagent binds VEGF with high affinity [32,33,34]. These results indicated that VEGF-Fn complexes are recognized by VEGFR2 and not by VEGFR1, suggesting that binding of VEGF to Fn may “activate” VEGF for VEGFR2 binding by shielding it from binding to its decoy receptor, VEGFR1.

### 2.4. Hypoxia Alters Heparan Sulfate Proteoglycans Expression

**Figure 6:**
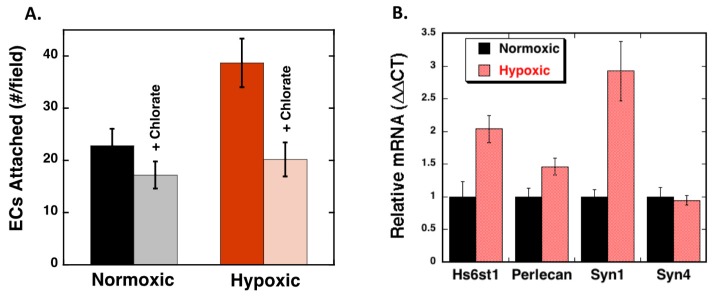
Hypoxia-mediated endothelial cell attachment correlates with increased HS proteoglycan expression. (**A**) RPE were seeded as described above and 50 mM sodium chlorate was added to the cell culture medium immediately prior to the onset of hypoxia. After 72 h, RECs were seeded onto the RPE cell layer and allowed to attach for 1 h and the counted (4 fields/well; 3 wells/condition; *n* = 4; *N* = 3). The data is expressed as the average number of cells per field ±SEM. (**B**) RPE cells were maintained under hypoxic (1% pO_2_) or normoxic (20% pO_2_) conditions for 72 h prior to total RNA extraction and mRNA quantitation using qPCR analyses. The data is normalized to the 18S RNA and to the normoxic control for each condition. The data is expressed as the average ±SEM (*N* = 4; *n* = 2). Hypoxia significantly increased REC attachment to RPE cells and chlorate caused a significant (*p* < 0.05) reduction in REC attachment to hypoxic cells, and hypoxia significantly increased expression of Hs6st1 and Syn1.

The increased deposition of VEGF in hypoxic cells suggest that the ECM under this condition is modified to enhance its capacity to bind VEGF. We have previously shown that ECM binding of VEGF involves a sophisticated interplay between HS and Fn whereby long HS chains with proper sulfation are able to enhance VEGF binding to Fn by altering the arrangement of the Hep 2 domain in Fn [16,21]. Thus, to determine if HS is likely involved in the observed hypoxia-mediated increase in endothelial cell attachment, RPE cells were treated with the biological sulfation inhibitor, sodium chlorate, during the normoxia/hypoxia treatment (Figure 6A). Chlorate treatment of RPE cells led to reduced REC adhesion to both normoxic and hypoxic cells, with the effect being much more dramatic with the hypoxic cells (24% versus 52% inhibition for normoxic and hypoxic cells, respectively). These results suggest that sulfated compounds, such as HS may be involved in enhanced cell adhesion caused by hypoxia.

To determine if hypoxia directly mediates HS expression, we measured RPE cell mRNA expression profiles of HS-proteoglycan core proteins and the enzyme that is involved in catalyzing the transfer of sulfate of the 6-*O* group on uronic acid residues (Hs6st1) after normoxia and hypoxia (Figure 6B). We have previously found that 6-*O* sulfation is critical for HS to be able to modify the Fn structure [16]. We observed significant increases in the expression of the HS modifying enzyme 6-*O*-sulfotransferase-1 (Hs6st1) and syndecan 1, a widely expressed HS proteoglycan (HSPG), under hypoxic conditions. RPE mRNA levels of perlecan were found to increase slightly with hypoxia, while syndecan 4 message levels were not changed as a consequence of hypoxia treatment. These findings suggest that hypoxia influences cellular HSPG production.

### 2.5. Heparan Sulfate Chains are Modified by Hypoxia

**Figure 7:**
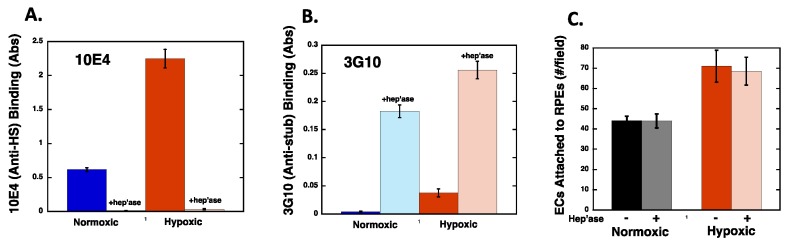
Heparan sulfate chain composition is altered by hypoxia. (**A**,**B**) RPE cells were maintained under normoxic or hypoxic (1% pO_2_) conditions for 72 h. During the last 2.5 h of culture, the cells were treated with or without 0.25 units/mL Heparinase III at 37 °C (hep’ase) prior to fixing. The cells were washed, fixed, blocked, and incubated with anti heparan sulfate antibodies clone 10E4 (**A**) or clone 3G10 (**B**) and HRP linked goat anti mouse IgG was used to detect 10E4 and 3G10. The data is expressed as blank corrected absorbance values corrected for cell number ±SEM where *N* = 4 for each condition. (**C**) After the hypoxia conditioning period and 2.5 h heparinase III treatment, RPE cells were washed and fluorescently labeled RECs allowed to attach for 1 h, washed, fixed, and counted. Data (4 fields/well; 3 wells/condition; *n* = 4; *N* = 3) is expressed as the average number of cells per field ±SD. Statistical analysis revealed that hypoxia significantly increased 10E4 and 3G10 binding to RPE cells (**A**,**B**), and that hypoxia increased REC attachment to RPE cells (**C**).

To further assess potential hypoxia-induced effects on HS expression, we used an in vitro ELISA system to identify relative changes in HS structures in cells maintained under normoxic and hypoxic conditions. For these studies, RPE were subject to normoxic or hypoxic conditions for 72 h and during the last 2.5 h of culture, the cells were treated in the absence or presence of Heparinase III prior to fixing. Fixed cell cultures were subject to ELISA using anti HS monoclonal antibodies, clones 10E4 and 3G10. The 10E4 antibody recognizes sulfate-rich epitopes present on native HS chains, while the 3G10 antibody recognizes a modified uronic acid stub left on HSPG core proteins following Heparinase III treatment [35]. Since there is only 1 HS stub present per HS chain generated in response to Heparinase III, reactivity of the 3G10 antibody directly reflects the number of HS chains expressed by a given cell type. As shown in Figure 7A, there was a dramatic increase (>3.5-fold) in 10E4 binding to RPE cells maintained under hypoxic conditions relative to RPE maintained under normoxic conditions. When both normoxic and hypoxic cells were treated with Heparinase III prior to fixing, 10E4 reactivity decreased significantly, which is consistent with the destruction of the HS epitope by the enzyme. As shown in Figure 7B, when Heparinase III-treated normoxic and hypoxic RPE cells were incubated with the 3G10 antibody, there was a modest difference in the absorbance profiles for normoxic and hypoxic RPE cell cultures (~40% increase with hypoxia). These data suggest that hypoxia did not dramatically alter the number of HS chains expressed by RPE cells, but did result in an alteration of chain length or general chemical structure, which resulted in more 10E4 epitopes present on each HS chain. Collectively, these results demonstrate that HS on RPE cells was chemically or structurally modified as a consequence of hypoxia. Interestingly there was no observed difference in REC attachment to the heparinase III treated RPE cells compared to the respective normoxic and hypoxic controls, indicating that HS is not used as a direct cell attachment site by the endothelial cells (Figure 7C). Instead these data are consistent with a role for HS in modulating Fn structure over the course of the hypoxia conditioning period.

## 3. Discussion

In this study we demonstrate that retinal endothelial cell attachment to RPE cells is enhanced by hypoxia as a result, in part, of increased VEGF expression and deposition in the ECM of RPE cells. Our data further indicate that this process is likely related to changes in HS expression and structure such that Fn within the matrix is available to bind VEGF and present it to VEGFR2 on endothelial cells. While these findings all derive from in vitro cell culture and cell-free assays, they nevertheless suggest interesting implications for regulation of angiogenesis in the retina and possibly other tissues.

Angiogenesis, the growth of new blood vessels from pre-existing ones, occurs throughout embryonic development and in a more restricted fashion in adults when tissues require additional sources of oxygen and nutrients [36,37,38,39]. When not properly regulated, angiogenesis can contribute to disease. In particular, pathologic angiogenesis in the retina is a major component of the progression of late-stage age-related macular degeneration (AMD) and proliferative diabetic retinopathy (PDR) [37,39,40]. AMD is the cause of greater than 50% of all new cases of blindness in the US, and diabetic retinopathy is the leading cause of blindness in working-age Americans [41,42,43]. With the combination of an aging population and the rapidly growing incidence of diabetes, both of these diseases are predicted to nearly double in the next decade, with estimates that 10–15 million Americans will suffer from one of these vision-robbing diseases by 2020 [43]. While these retinopathies have clear distinctions, they have in common an association with pathological angiogenesis that is driven by hypoxia and the potent angiogenic factor VEGF. Excessive VEGF can promote choroidal vessel invasion through Bruch’s membrane into the retina (AMD) as well as intraretinal neovascularization (PDR), and in both instances incompetent blood vessels lead to hemorrhage, scarring, inflammation, and macular edema [37,40,44]. Indeed, anti-VEGF treatments have shown great promise in slowing the progression of late-stage AMD and recent trials suggest that these approaches may also be effective for treatment of PDR [45,46,47]. However, these current treatments remain limited and do not halt or reverse disease. This is likely a reflection of inefficient pharmacokinetics/dynamics of the current drugs as well as the fact that our basic understanding of the complex mechanisms controlling angiogenesis remains incomplete.

Deposition of VEGF within the ECM is dependent on available binding sites on Fn, which are controlled by the presence of HS of proper structure for Fn activation. Thus, it is possible that conditional activation of HS may play important roles in disease and injury. Interestingly, a strong correlation has been observed between CNV and increased expression of HS proteoglycans in the retina in rat models, suggesting that HS contributes to pathologic angiogenesis [48]. Consistent with this concept, intravitreal injection of a soluble HS mimetic has been shown to inhibit laser-induced CNV in rats [49]. Together with our older observation that heparinases, enzymes that degrade HS, can inhibit angiogenesis [50], these findings suggest that endogenous HS plays critical roles in mediating neovascularization in the retina. In this present study, we found that hypoxia modulates the biosynthetic machinery to potentially lead to increased levels of active HS that can drive VEGF deposition within a Fn-rich ECM. Moreover, the increased ECM-localized VEGF might selectively bind to VEGFR2 on endothelial cells, thus, promoting angiogenesis.

Hypoxia is a well-known inducer of angiogenesis [28,29,51,52,53]. Indeed, several previous studies have also found a link between hypoxia and ECM remodeling [54,55,56]. Hypoxia-induced alterations include changes in composition and mechanical properties, namely ECM stiffness. We conducted the present studies on tissue culture plastic, which is a non-physiologically stiff substrate, especially compared to the retina, thus, it would be interesting to explore if hypoxia also modulates the stiffness of the RPE ECM by culturing cells on softer gels. Indeed, a recent study of corneal epithelial cells noted significant differences in the response of these cells to hypoxia based on the stiffness of the matrix used [55]. This is interesting in light of our previous work showing that VEGF responsiveness of endothelial cells is modulated by matrix stiffness [57,58] and that the ability of heparin and heparan sulfate to regulate Fn structure, and VEGF binding site availability, is responsive to mechanical force applied to the Fn fibers [59]. Thus, while we show interesting alterations in endothelial cell attachment and ECM composition in response to hypoxia, it is important to recognize that a complete understanding of how cells respond to hypoxia will require a more thorough evaluation of the inter-play of other environmental factors.

**Figure 8:**
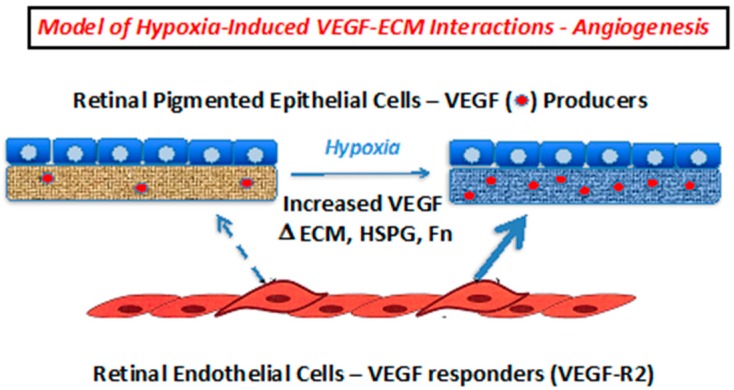
Proposed model for paracrine control of choroidal neovascularization via alterations in VEGF deposition within the RPE ECM. Hypoxia results in increased VEGF expression, and changes in the capacity of the RPE ECM to bind to VEGF. The increased VEGF-laden matrix may then selectively bind to VEGFR2 on endothelial and endothelial progenitor cells to facilitate the interaction with the ECM and ultimately, angiogenesis.

The ability of cells to recognize and respond effectively to alterations in the extracellular environment is key to maintenance and regeneration of healthy tissue. We discovered a novel mechanism whereby heparin/HS can catalytically modulate an Fn structure, leading to exposure of a cryptic binding site for VEGF [21,22]. This activity requires long (> 22 units) oligosaccharide chains containing 6-*O* and *N*-sulfation [16]. Here we note alterations in HS produced by RPE cells subjected to hypoxia. Thus, the increase in HS chain length/structure correlated with increased Hs6st1 mRNA expression in hypoxic RPE cells may indicate a mechanism for switching HS from a catalytically inactive to active state, thus allowing modification of Fn within the ECM leading to VEGF deposition and recruitment and activation of new blood vessel growth. These findings suggest an intriguing paracrine mechanism that might contribute to choroidal neovascularization in the retina, whereby hypoxic conditions modulate the ECM to prime it for recruiting endothelial cells (Figure 8). This information adds to the understanding of how angiogenesis is controlled in the retina, and likely other tissues, and may provide valuable new insight toward the development of more effective therapies for diseases associated with pathological angiogenesis.

## 4. Materials and Methods

### 4.1. Reagents

All cell culture plates and general-purpose reagents were obtained from Corning Cellgro (Manassas, VA, USA). Human recombinant VEGF-_165_ was obtained from the NCI Bulk Cytokine and Monoclonal Antibody Preclinical Repository (Frederick, MA, USA). ^125^I-VEGF_165_ was prepared using Bolton–Hunter reagent obtained from Perkin Elmer (Boston, MA, USA). Human plasma fibronectin was obtained from Millipore (Temecula, CA, USA). Vybrant Cell Labeling Solutions were purchased from In Vitrogen Molecular Probes (Eugene, OR, USA). Monoclonal antibodies to heparan sulfate (clones 3G10 and 10E4) were obtained from Northstar BioProducts (East Falmouth, MA, USA) and the HRP linked anti-IgG secondary antibodies were purchased from Jackson ImmunoResearch Laboratories (West Grove, PA, USA). Heparinase III (E.C. 4.2.2.8) was from Biomarin Pharmaceuticals (Montreal, Canada). Recombinant Fc-VEGF Receptor 1 (#321-FL) and Fc-VEGF Receptor 2 (#357-KD) chimera proteins containing the extracellular domain of the human receptors were purchased from R & D Systems (Minneapolis, MN, USA). DNAse I was obtained from New England Biolabs (Beverly, MA, USA) and the RNAse I inhibitor was obtained from Life Technologies (Grand Island, NY, USA). All other chemicals were reagent grade products obtained from commercial sources.

### 4.2. Cell Culture

Retinal pigmented epithelial (RPE) cells from human were generously provided by Dr. Nader Rahimi and were maintained in Dulbecco’s modified Eagle’s medium (DMEM) (Corning, Tewkesbury, MA) containing 10% calf serum (Hyclone, Logan UT, USA), 2 mM *L*-glutamine, and 100 units/mL penicillin G and 100 µg/mL streptomycin (Corning, Tewkesbury, MA). Rat Retinal Endothelial cells (REC), generously provided by Dr. Sayon Roy, and vascular smooth muscle cells, isolated from neonatal rat aortas as described [60], were maintained in DMEM containing 10% fetal bovine serum (Atlanta Biologicals, Lawrenceville, GA, USA), 2 mM *L*-glutamine, and 100 units/mL penicillin G and 100 µg/mL streptomycin. Hypoxia conditions were induced by maintaining the cells in an environment composed of 1% pO_2_ in a Galaxy 48R CO_2_ Incubator (Eppendorf North America, Hauppague, NY, USA) for the indicated treatment times while normoxic cells were maintained in a humidified cell culture incubator at 20% pO_2_.

### 4.3. Cell Attachment Assays

**Figure 9:**
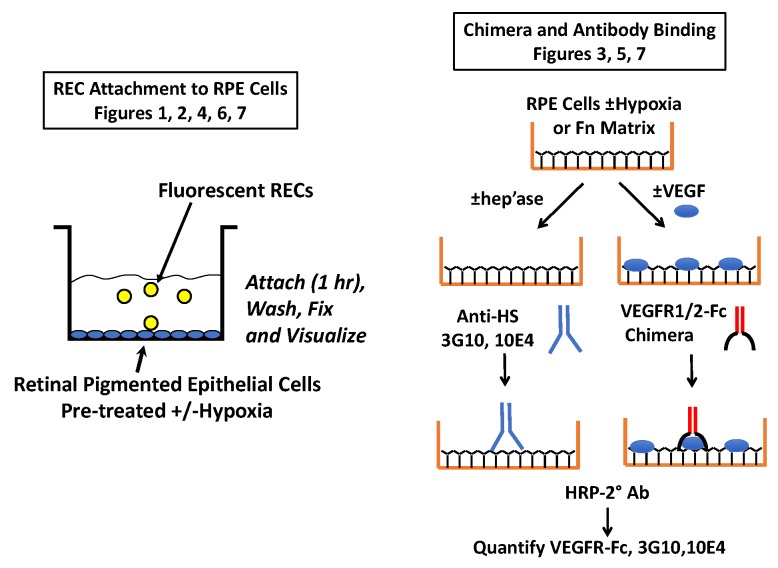
Schematic of key methods. On the left is the general cell attachment protocol. Fluorescent RECs were allowed to attach to RPEs that had been preconditioned ± hypoxia. In some studies, soluble antagonists were added with the RECs and in others the cells were treated prior to REC attachment (i.e., with VEGF or heparinase III). On the right is the general approach used to evaluate VEGF-receptor binding to RPEs and Fn, as well as the method used to analyze HS levels on RPEs.

RPE cells were seeded at 6.5 × 10^4^ cells per well (2 cm^2^) and maintained overnight prior to initiating hypoxia treatment for an additional 48–72 h (Figure 9). RECs were collected using trypsin and were resuspended in serum free DMEM and labeled using the Vybrant DiO cell labeling solution according to the manufacturer’s recommendations. Fluorescently labeled REC (50,000 cells/cm^2^) were added to normoxic and hypoxic RPE cell layers and were allowed to attach for 1 h at 37 °C. Unattached cells were removed by washing the cell layers with PBS three times prior to fixation using 4% paraformaldehyde solution for 20 min on ice. The cell layers were washed with PBS and stored at 4 °C prior to visualization using a Nikon Eclipse TE 200 fluorescent microscope with SPOT imaging camera. In studies that utilized sodium chlorate, the cells were seeded as described above and were maintained overnight prior to the addition of 50 mM sodium chlorate and then maintenance in hypoxic/normoxic conditions for the indicated time. For studies utilizing the various inhibitors, the cells were plated and were maintained overnight as described above and 100 ng/mL Fc-VEGF Receptor 2 chimera protein, 10 µg/mL Fibronectin 40 kD Hep 2 domain (sFn), or 100 µM sucrose octasulfate (SOS) was added to the washed RPE cells immediately prior to adding the labeled REC cells.

### 4.4. Real Time Polymerase Chain Reaction (RT-PCR) Analyses

Total RNA was isolated from RPE cells using guanidinium thiocynate extraction as described previously [61]. Genomic DNA was removed by incubation with DNAse I in the presence of an RNAse inhibitor. cDNA synthesis was carried out by annealing the RNA with random hexamer and oligo dT primers and first strand synthesis was conducted using MuLV reverse transcriptase. Negative control runs were performed in the absence of reverse transcriptase. RT-PCR analysis was carried out using a 7300 Real Time PCR system (Applied Biosystems, Foster City, CA, USA). ABI TaqMan gene expression analysis was conducted with the indicated target probes from Applied Biosystems, as follows: Syndecan 1 (Hs00896424_g1), syndecan 4 (Hs01120908_m1), perlecan (Hs01078536_m1), Hs6St1 (Hs00757137_m1), and eukaryotic 18S rRNA (4308329) was used as the internal control. Cycle number for 18S rRNA expansion did not vary with time or treatment indicating stability of this endogenous control, and control studies done in previous studies confirm the stability of 18S rRNA from a variety of cultured cells.

### 4.5. Proteoglycan ELISAs

For heparan sulfate ELISAs, RPE were seeded into 96 well plates at a density of 25,000 cells/cm^2^ and were maintained overnight. The cells were incubated under normoxic (20% pO_2_) or hypoxic (1% pO_2_) conditions for an additional 72 h. For the last 2.5 h of culture, the cells were treated without or with 0.25 units/mL Heparinase III (E.C. 4.2.2.8) prior to washing and fixing with 4% formaldehyde solution. The wells were blocked overnight with 3% BSA-TBS and were washed with tris buffered saline (50 mM tris pH 7.4, 150 mM sodium chloride; TBS) prior to incubation with antibodies against native or heparinase III modified heparan sulfate. The cells were incubated with 0.66 µg/mL clone 3G10 or 1.33 µg/mL clone 10E4 in blocking solution for 90 min at room temperature. The wells were washed with TBS prior to incubation with 0.32 µg/mL HRP conjugated goat-anti-mouse IgG for 30 min. The wells were washed with TBS containing 0.1% tween 20 and TBS prior to development using the TMB 2 Component Microwell Peroxidase Substrate System (KPL, Gaithersburg, MD, USA). The reaction was stopped with the addition of 1N sulfuric acid and the wells were incubated briefly prior to reading the absorbance at 450 nm and 570 nm (background correction). To account for differences in cell numbers between normoxic and hypoxic cell cultures, relative cell numbers were determined using the acid phosphatase assay [62]. The data is expressed as the average blank corrected absorbance values corrected for cell number ± SEM.

### 4.6. VEGF Receptor Chimera Assays

Polystyrene wells were coated with 1 µg (3.125 µg/cm^2^) fibronectin and 1µg heparin overnight at 4 °C. The solution was removed and the wells were washed, removing the heparin, and were incubated with 0–250 ng/well VEGF in Binding Buffer pH 6.5 (DMEM, 1 mg/mL BSA, 25 mM HEPES pH 6.5) for 1 h on ice at 4 °C. The wells were washed and incubated with 25 ng/well Fc-VEGFR1 or Fc-VEGFR2 chimera proteins for 1 h at room temperature. The wells were washed and incubated with a 1:5000 dilution of HRP-linked goat anti human IgG for 30 min at room temperature. The wells were aspirated and washed prior to development using TMB substrate reagent. The color development was stopped using 1N sulfuric acid and the absorbance was read at 450 and 570 nm (background) using an OPTImax microtiter platereader (Molecular Devices Corporation, Sunnyvale, CA, USA). For cell-based experiments, RPE cells were seeded and maintained under normoxic or hypoxic conditions for 72 h prior to assay. The cells were incubated with or without VEGF and were processed using the experimental conditions described above.

### 4.7. VEGF Binding Assays

RPE cells were seeded into 48 well plates at 20,000 cells per well. The cells were maintained overnight prior to placing the cells into the hypoxia chamber or maintaining the cells under normoxic conditions. The cells were subjected to hypoxia/normoxia for 72 h prior to conducting binding assays. For binding assays, the medium was removed and the cells were washed 3 times with ice cold binding buffer (25 mM Hepes pH 6.5, 1 mg/mL BSA, DMEM; 200 µL/well). A total of 100 µL of binding buffer was added to each well and the cells were incubated at 4 °C for 10 min. ^125^I-VEGF (20 ng/mL) was added to each well and allowed to bind to the cells for 2 h at 4 °C, until equilibrium was reached. Unbound ^125^I-VEGF was removed by washing the cells 3 times with ice cold binding buffer and the ^125^I-VEGF that was bound to fibronectin and HSPG was released by exposing the cells to high salt buffer (25 mM Hepes, 2 M NaCl; 100 µL/well) for 5 s, followed by PBS (100 µL/well), and the radioactivity in the combined samples was counted in a Packard Instruments Auto-gamma counter.

### 4.8. Statistical Analysis

Statistical significance of data was evaluated using analysis of variance (ANOVA) followed by the Tukey’s multiple-comparisons test, where appropriate, using GraphPad Prism version 6.0 (San Diego, CA, USA). A two-tailed *t*-test was conducted when only one variable was evaluated (e.g., Figure 1). Differences were considered significant when *p* values were < 0.05. Data are presented as the means of replicate samples from a selected experiment ± SD or SEM as indicated in the legends to figures.

## 5. Conclusions

Hypoxia enhances endothelial cell attachment to RPE cells through modifications in the ECM that allow increased VEGF deposition, which in turn, is presented to VEGFR2, and shielded from VEGFR1. This mechanism might be an important component for how hypoxia stimulates the growth of new blood vessels to under-vascularized tissues.

## Figures and Tables

**Table 1:** Summary of VEGF-ECM Antagonists.

Reagent	Mode of Action	Expected Impact
Fc-VEGF-R2	Binds to VEGF in the ECM.	Block VEGF-R2 binding site on VEGF. Specifically prevents VEGF-R2 on RECs from engaging VEGF in the ECM.
sFn	Competes for VEGF specifically bound to Fn.	Binds and releases VEGF from Fn and into soluble VEGF-sFn form. Reduces sites for VEGF-R2 on RECs to bind in the ECM.
SOS	Binds to VEGF and blocks its binding to Fn.	Reduces VEGF-Fn complexes. Reduces sites for VEGF-R2 on RECs to bind in the ECM.

## Abbreviations

RPE	Retinal pigmented epithelial cells
REC	Rat retinal endothelial cells
VEGF	Vascular endothelial growth factor-A165
Fc-VEGFR1	The binding domain of VEGF receptor 1 linked to the fragment crystallizable tail region of human antibody.
Fc-VEGFR2	The binding domain of VEGF receptor 2 linked to the fragment crystallizable tail region of human antibody.
Hs6st1	Heparan sulfate specific 6-O sulfoltransferase-1
Syn1	Syndecan 1
Perl	Perlecan
Syn4	Syndecan 4
PBS	Phosphate buffered saline
BSA	Bovine serum albumin
AMD	Age-related macular degeneration
PDR	Proliferative diabetic retinopathy
HRP	Horse radish peroxidase
EC	Endothelial cells
HS	Heparan sulfate
HSPG	Heparan sulfate proteoglycans
N	Biological replicates
n	Technical replicates
